# Ticagrelor vs. Clopidogrel in Older Patients With Acute Coronary Syndrome Undergoing Percutaneous Coronary Intervention: Insights From a Real-World Registry

**DOI:** 10.3389/fcvm.2022.859962

**Published:** 2022-03-15

**Authors:** Yunnan Zhang, Wenxing Peng, Xiujin Shi, Jialun Han, Yifan Wang, Zhenwei Fang, Yang Lin

**Affiliations:** ^1^Department of Pharmacy, Beijing Anzhen Hospital, Capital Medical University, Beijing, China; ^2^School of Pharmaceutical Sciences, Capital Medical University, Beijing, China

**Keywords:** clopidogrel, ticagrelor, antiplatelet therapy, acute coronary syndrome, older patients

## Abstract

**Background and Objectives:**

It is unclear whether more potent P2Y12 inhibitors are of benefit to older patients who are at high risk for both ischemia and bleeding. We conducted an observational study to compare the clinical outcomes of clopidogrel and ticagrelor uses in older patients with an acute coronary syndrome (ACS).

**Methods:**

Older patients (aged ≥65 years) with ACS who underwent percutaneous coronary intervention (PCI) were divided into clopidogrel-treated and ticagrelor-treated groups. The primary observational endpoint was the occurrence of net adverse clinical and cerebral events (NACCEs) during a 12-month period, which is defined as the composite endpoint of all-cause death, myocardial infarction (MI), stroke, stent thrombosis, urgent coronary revascularization, and clinically significant bleeding. The secondary endpoints were clinically significant bleeding and major adverse clinical and cerebral events (MACCEs).

**Results:**

This study included a total of 2,611 patients. Of them, 1,636 received clopidogrel and 975 received ticagrelor. Between patients receiving clopidogrel and those receiving ticagrelor, no significant differences were noted in NACCE (8.4 vs. 9.7%, respectively; adjusted hazard ratio [HR], 0.86; 95% confidence interval [CI], 0.66–1.12) or MACCE (7.1 vs. 7.0%, respectively; adjusted HR, 1.13; 95% CI, 0.83–1.55) during the 12-month follow-up period. In contrast, the occurrence of clinically significant bleeding was significantly less in clopidogrel-treated patients compared with that in ticagrelor-treated patients (27, 1.7%, vs. 31, 3.2%, respectively; adjusted HR, 0.42; 95% CI, 0.25–0.69). Stratified analyses revealed no significant association between age (≥75 years vs. <75 years) and treatment condition in terms of primary or secondary endpoints.

**Conclusion:**

This study showed that clopidogrel and ticagrelor had comparable net clinical benefits in patients with ACS aged ≥65 years. Additionally, clopidogrel was associated with a significantly lower risk of major bleeding than ticagrelor without an increase in ischemic risk. These findings suggest that clopidogrel is an effective alternative to the more potent P2Y12 inhibitor ticagrelor in older patients.

## Introduction

Dual antiplatelet therapy (DAPT), which comprises a P2Y12 inhibitor and aspirin, is the standard antiplatelet therapy strategy in patients with acute coronary syndrome (ACS) who are undergoing percutaneous coronary intervention (PCI). Since two landmark studies (1, 2) confirmed that the potent P2Y12 inhibitors prasugrel and ticagrelor are superior to clopidogrel in reducing ischemic events, European and American guidelines (3, 4) have recommended potent P2Y12 inhibitors in patients with ACS as antiplatelet therapy, regardless of age. However, advanced age is an important predictor of adverse clinical outcomes after ACS (5). Older patients usually have higher risks of ischemic and also bleeding events related to antiplatelet drugs (6). Cardiologists should exercise caution in balancing bleeding and ischemic risks in older patients with ACS while formulating an optimal DAPT strategy.

Several previous studies have compared the clinical outcomes of using clopidogrel and potent antiplatelet inhibitors in older patients with ACS; however, the outcomes have not yet been fully determined. A substudy of the PLATelet inhibition and patient Outcomes (PLATO) trial (7) showed that ticagrelor was superior to clopidogrel in reducing the risk of the primary composite outcome of cardiovascular death, myocardial infarction (MI), or stroke with no age–treatment associations (≥75 years vs. <75 years; *p* = 0.56). However, a recently published randomized non-inferiority trial (POPular AGE) demonstrated that clopidogrel is a favorable alternative P2Y12 inhibitor to ticagrelor in patients aged ≥70 years, particularly in those with high-bleeding risk, because it reduces the bleeding risk without increasing ischemic events (8). Additionally, a previous study in patients with ACS with high-bleeding risk showed that clopidogrel and ticagrelor were comparable in terms of ischemia risk reduction (9). The abovementioned evidence presents a challenge to the use of potent P2Y12 inhibitors in older patients. However, most of the existing studies have been conducted in Caucasian patients; studies that compare the use of clopidogrel and ticagrelor in older Asian patients are worth conducting.

A further potential confounding factor is the variable definition of older individuals, which can contribute to inconsistent conclusions. Although a common definition considers individuals aged >75 years as older adults, a cutoff of 65 years is also applied in age and risk stratification (5, 6, 10, 11). Considering the abovementioned background and to address the need for an optimal DAPT strategy in older patients, we conducted the present retrospective observational cohort study by comparing the clinical outcomes of clopidogrel and ticagrelor uses in patients with ACS aged ≥65 years who underwent PCI.

## Methods

### Study Design and Participants

All participants were recruited from the PHARM-ACS registry (NCT04184583). PHARM-ACS is an ambispective single-center registry study conducted at Beijing Anzhen Hospital, China, to investigate pharmacotherapy and its long-term clinical outcomes in patients with ACS. Patients were recruited retrospectively from April 2018 to November 2019 and prospectively after December 2019 if they (1) were aged ≥18 years; (2) were diagnosed with ACS, including ST segment elevation MI (STEMI), non-STEMI, or unstable angina; (3) agreed to sign an informed consent form; and (4) had a life expectancy of ≥12 months. The main exclusion criteria were as follows: (1) missing data such as data related to medical history and demographics; (2) pregnancy or lactation status; or (3) severe mental disorders that prevent compliance with the study protocol. The diagnostic criteria for ACS were per the guidelines for the diagnosis and treatment of non-ST segment elevation ACS and STEMI (12, 13). The study protocol was approved by the Ethics Committee of Beijing Anzhen Hospital, and patient privacy was guaranteed throughout the study. A specially established electronic data capture system was used in this registry. All available data on demographics, comorbidities, procedures performed, medications used, and follow-up were converted to a standardized format and were then uploaded to the data capture system.

All patients from the PHARM-ACS registry who met the following criteria were considered eligible: patients were (1) aged ≥65 years, (2) underwent successful PCI, (3) received clopidogrel or ticagrelor plus aspirin at discharge, and (4) were discharged before November 2020 to allow for at least 1 year of follow-up. The primary exclusion criteria were as follows: (1) the use of other antiplatelet drugs such as cilostazol at discharge, (2) long-term treatment with oral anticoagulants, (3) change in or termination of antiplatelet drug use within 1 year after discharge, and (4) death during hospitalization because of reasons other than stent thrombosis. Eligible patients were divided into clopidogrel-treated and ticagrelor-treated groups. Because this was an observational study, the selection of P2Y12 inhibitors was at the discretion of cardiologists.

### Study Endpoints

The primary study endpoint was the occurrence of net adverse clinical and cerebral events (NACCEs), which is defined as the composite endpoint of all-cause death, MI, stroke, stent thrombosis, urgent coronary revascularization, and clinically significant bleeding (Bleeding Academic Research Consortium class [BARC] ≥2). MI was defined as elevated myocardial necrosis biomarkers (creatine kinase-MB or troponin) with at least one of the following indicators: myocardial ischemia symptoms, ischemic electrocardiography (ECG) changes (ST segment, T wave, or new left bundle branch block), or pathological Q wave on ECG (12, 13). Stroke was defined as acute neurological deficit due to vascular dysfunction of the central nervous system. Stent thrombosis was defined as partial or complete thrombotic occlusion around the stent area confirmed using vascular imaging or pathology analysis, with at least one of the following indicators: acute ischemic symptoms, ischemic ECG changes, or elevated myocardial necrosis biomarkers. Urgent coronary revascularization was defined as unplanned coronary revascularization due to ACS. Clinically significant bleeding was defined as BARC class ≥2 as mentioned above (14). The secondary endpoints were clinically significant bleeding and major adverse clinical and cerebral events (MACCEs). The events were determined based on the diagnostic reports derived from the electronic medical record system or provided by the patients. The identification of clinically significant bleeding events also included self-reported information of the patients. All events were determined by at least two cardiologists.

### Follow-Up

Regular follow-ups were conducted every 6 months by uniformly trained medical staff to ensure standard procedures. Follow-up methods included telephone interviews, clinic visits, and WeChat messaging. A standard case report form was used during each follow-up interview to obtain information on clinical outcomes, medication use, and other drug-related adverse events.

### Statistical Analysis

Statistical analysis was performed using R studio (version 1.4). Continuous variables were described as means ± standard deviations or medians (interquartile ranges [IQRs]) and compared using student's *t*-test or a non-parametric test based on whether the data conformed to a normal distribution. Categorical variables were reported as frequencies (percentages) and compared using chi-square test. Cox proportional-hazards regression models were used to calculate the hazard ratios (HRs) and 95% CIs, to compare the primary and secondary outcomes between the two groups. Because the division of patients into the two groups was not random, baseline characteristics varied significantly between the two groups. Two types of Cox regression models were considered to obtain adjusted HR values: the (1) multivariate Cox regression model and (2) inverse probability of treatment weighting (IPTW)-adjusted Cox regression model. Stabilized weights were used to avoid high variability (15). Standardized differences of ≤ 0.10 indicated well-balanced covariates while using IPTW. Covariates used in these two models were selected based on the previous studies and were reported to be related to clinical outcomes (16–18). The final models included age, sex, body mass index (BMI), smoking status, PCI indication, PCI type, stent number, multiple-vessel disease, medical history (previous PCI with stenting, previous coronary artery bypass grafting, hypertension, hyperlipidemia, diabetes mellitus, MI, heart failure, and cerebral infarction), left ventricular ejection fraction, estimated glomerular filtration rate, and discharge medication (statin, angiotensin-converting enzyme inhibitor or angiotensin receptor blocker, beta blocker, and proton pump inhibitor). The cumulative event rates of the primary and secondary outcomes within a 12-month period were weighted using IPTW values and plotted as Kaplan–Meier curves. Stratified analyses were performed to determine the interactions between the treatment condition and age (≥75 years and <75 years), sex, BMI (>30 and ≤ 30 kg/m^2^), or diabetes mellitus. *p* < 0.05 were considered statistically significant.

## Results

**Figure 1:**
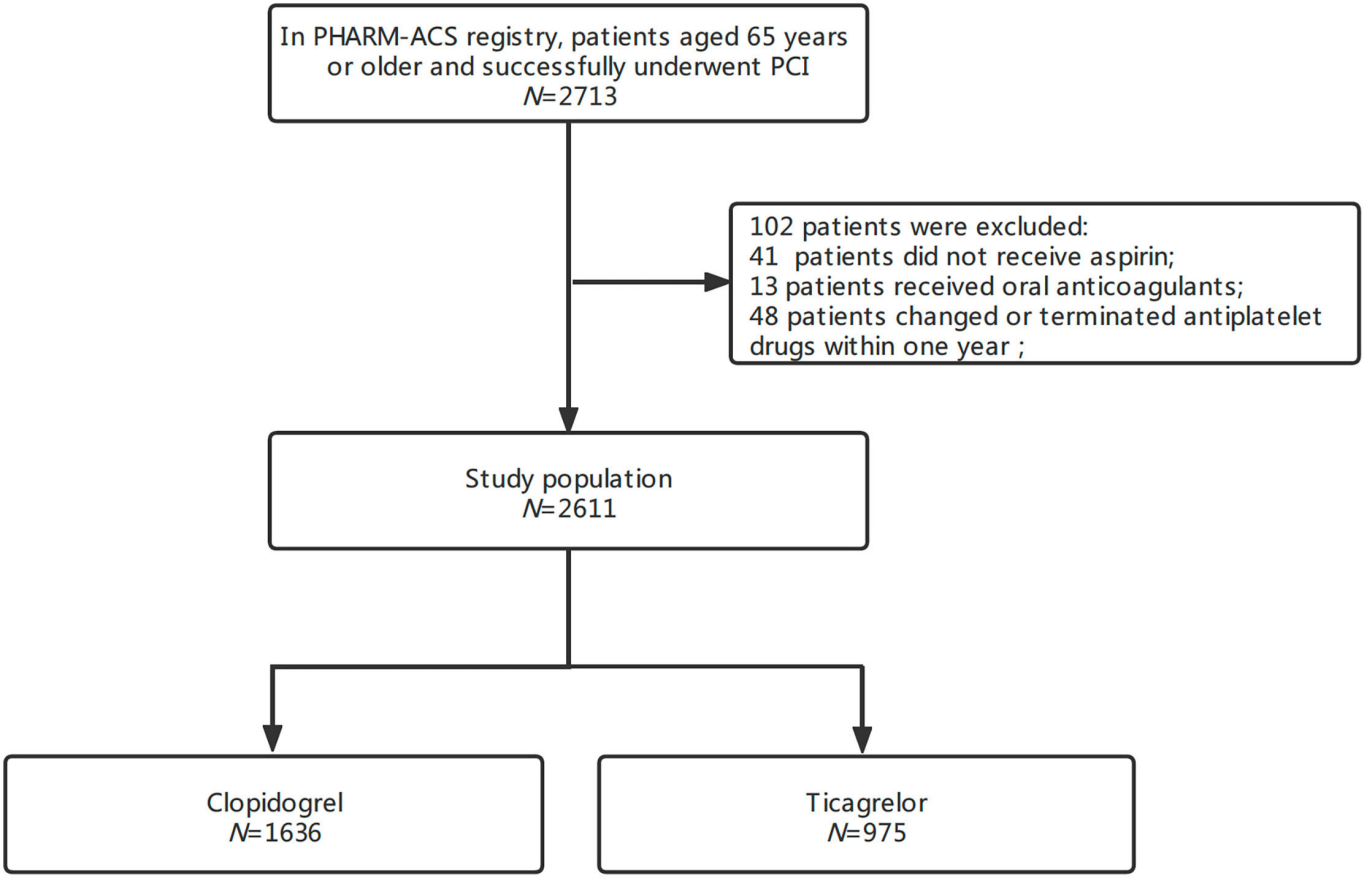
Study flowchart. ACS, acute coronary syndrome; PCI, percutaneous coronary intervention.

In the PHARM-ACS registry, a total of 2,713 patients aged ≥65 years had ACS and underwent PCI. A total of 102 patients were excluded because of not receiving aspirin (41), receiving oral anticoagulants (13), or changing or terminating antiplatelet drugs (48) within 1 year. Finally, 2,611 patients were enrolled in this study (Figure 1). The average age was 70.3 ± 4.5 (range, 65–90) years; 63.3% were men and 30.3% had undergone previous PCI with stenting. Among them, 1,636 patients (62.7%) received clopidogrel and 975 (37.3%) received ticagrelor. The median follow-up time was 538 (IQR 449–647) days. There were several differences in baseline characteristics between the two groups (*p* < 0.05). The raw data showed compared with clopidogrel-treated patients, ticagrelor-treated patients had lower average age, higher proportion of men, differences in the occurrence of STEMI, previous PCI with stenting, multiple-vessel diseases, and hypertension (Table 1). After IPTW adjustment, the standardized differences in all covariates were below 0.10, which suggests well-normalized differences between the two groups.

**Table 1:** Patients' demographic and clinical factors before and after IPTW.

**Characteristics**	**Before IPTW**			**After IPTW**		
	**Clopidogrel**	**Ticagrelor**	**Standardized difference**	**Clopidogrel**	**Ticagrelor**	**Standardized difference**
	***N** **=*** **1,636 (%)**	***N** **=*** **975 (%)**		***N** **=*** **1,636 (%)**	***N** **=*** **976 (%)**	
**Age**	70.9 ± 4.8	69.3 ± 3.9^*^	0.38	70.3 ± 4.6	70.2 ± 4.4	0.02
≥75 years	370 (22.6)	107 (11.0)^*^	0.31	306 (18.7)	164 (16.8)	0.05
**Male**	990 (60.5)	663 (68.0)^*^	0.16	1,036 (63.3)	612 (62.7)	0.01
**Body mass index^a^**	25.8 ± 10.9	26.0 ± 14.3	0.02	26.0 ± 12.6	26.3 ± 16.8	0.02
**Current smoker**	295 (18.0)	204 (20.9)	0.07	309 (18.9)	181 (18.5)	0.01
**PCI indication**						
STEMI	83 (5.1)	116 (11.9)^*^	0.25	119 (7.3)	80 (8.2)	0.04
Non-STEMI	159 (9.7)	101 (10.4)	0.02	177 (10.8)	81 (8.3)	0.08
Unstable angina	1,394 (85.2)	758 (77.7)^*^	0.19	1,340 (81.9)	815 (83.5)	0.04
**PCI type**						
Drug-eluting stent	1,475 (90.2)	909 (93.2)^*^	0.11	1,485 (90.8)	907(92.9)	0.07
Balloon angioplasty	161 (9.8)	66 (6.8)^*^	0.11	151 (9.2)	69 (7.1)	0.07
**No. of stent**						
≤ 1	986 (60.3)	504 (51.7)^*^	0.10	956 (58.4)	536 (54.9)	0.07
>1, <3	432 (26.4)	275 (28.2)	0.04	436 (26.7)	270 (27.7)	0.02
≥3	218 (13.3)	196 (20.1)^*^	0.18	244 (14.9)	170 (17.4)	0.07
**Multiple-vessel disease**	256 (15.6)	225 (23.1)^*^	0.19	290 (17.7)	203 (20.8)	0.08
**Medical history**						
Previous PCI with stenting	465 (28.4)	326 (33.4)^*^	0.11	493 (30.1)	288 (29.5)	0.01
Previous CABG	49 (3.0)	30 (3.1)	0.01	49 (3.0)	31 (3.2)	0.01
Hypertension	1,184 (72.4)	647 (66.4)^*^	0.13	1,144 (69.9)	683 (69.9)	0.00
Hyperlipidemia	1,158 (70.8)	713 (73.1)	0.05	1,164 (71.1)	683 (70.0)	0.03
Diabetes mellitus	613 (37.5)	360 (36.9)	0.01	610 (37.3)	366 (37.5)	0.00
Myocardial infarction	178 (10.9)	123 (12.6)	0.05	197 (12.0)	118 (12.1)	0.00
Atrial fibrillation	44 (2.7)	16 (1.6)	0.08	37 (2.3)	18 (1.8)	0.03
Heart failure	18 (1.1)	9 (0.9)	0.02	17 (1.0)	10 (1.0)	0.01
Cerebral infarction	197 (12.0)	77 (7.9)^*^	0.14	171 (10.4)	100 (10.3)	0.01
**Left ventricular EF%^a^**	61.7 ± 7.6	60.6 ± 8.7^*^	0.14	61.7 ± 7.5	61.3 ± 8.3	0.05
**eGFR^a^**	78.4 ± 21.8	81.2 ± 21.1^*^	0.13	79.2 ± 21.8	80.1 ± 21.3	0.04
**Discharge medication**						
Statin	1,605 (98.1)	950 (97.4)	0.05	1,600 (97.8)	957 (98.0)	0.01
ACEI or ARB	657 (40.2)	393 (40.3)	0.00	656 (40.1)	388 (39.7)	0.01
Beta blocker	1,038 (63.4)	667 (68.4)^*^	0.11	1,066 (65.2)	635 (65.1)	0.00
Proton pump inhibitor	1405 (85.9)	803 (82.4)^*^	0.10	1378 (84.2)	822 (84.2)	0.00

*ACEI, angiotensin-converting enzyme inhibitor; ARB, angiotensin receptor blocker; CABG, coronary artery bypass grafting; eGFR, estimate glomerular filtration rate; EF, ejection fraction; IPTW, inverse probability treatment weighting; PCI, percutaneous coronary intervention; STEMI, ST segment elevation MI. Standardized difference>0.10 indicates that the variable is not balanced between the two groups*.

a *There are 53 missing data in BMI, 76 missing data in eGFR, and 212 missing data in left ventricular EF. The eGFR values were calculated based on the Modification of Diet in Renal Disease (MDRD) formula*.

* *p < 0.05 compared with the ticagrelor group*.

**Figure 2:**
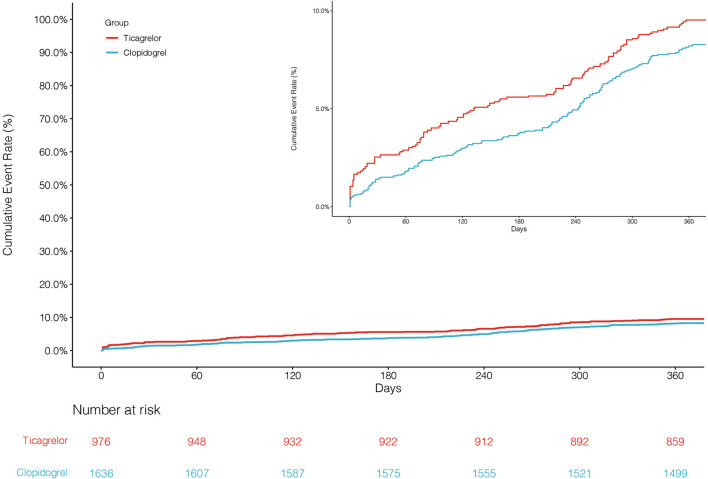
Cumulative incidence of NACCEs after inverse probability of treatment weights.

**Figure 3:**
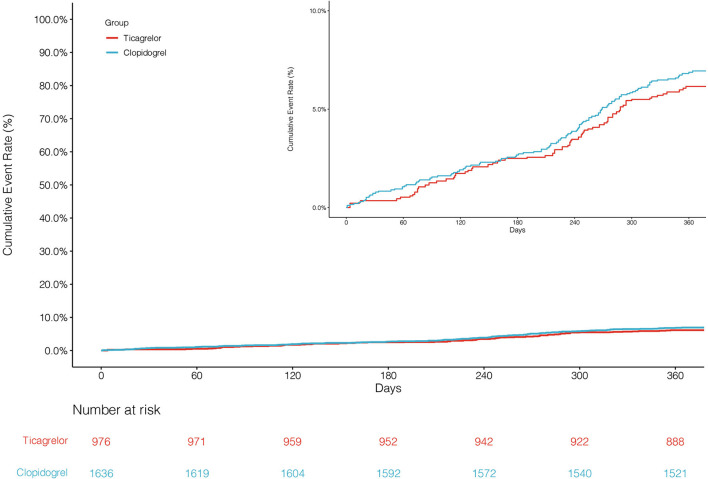
Cumulative incidence of MACCEs after inverse probability of treatment weights.

**Figure 4:**
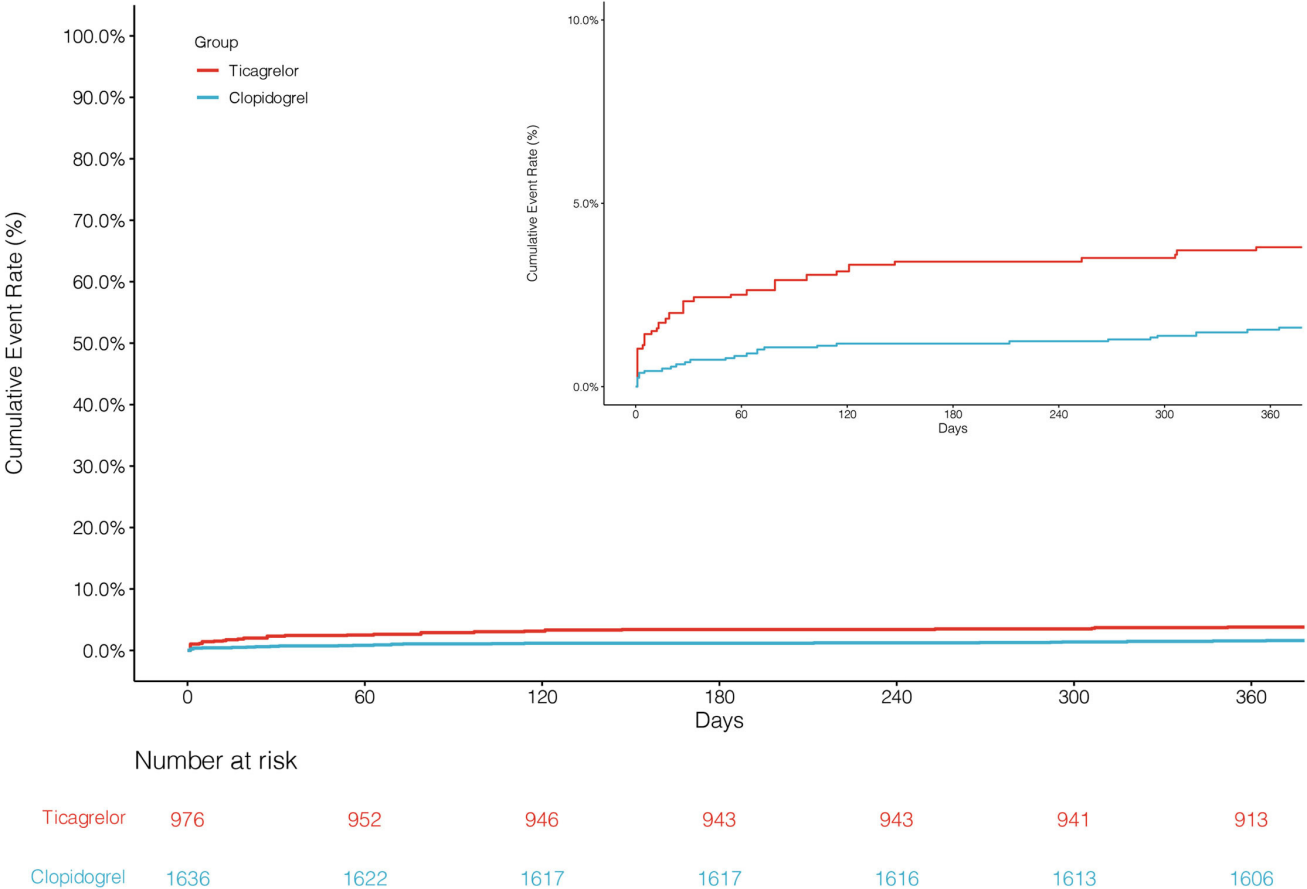
Cumulative incidence of clinically significant bleeding after inverse probability of treatment weights.

The primary endpoint NACCE occurred in 138 (8.4%) clopidogrel-treated patients and 95 (9.7%) ticagrelor-treated patients. No significant difference was found in NACCE between the two groups (unadjusted HR, 0.86, and 95% CI, 0.66–1.11; IPTW-adjusted HR, 0.86, and 95% CI, 0.66–1.12; Table 2; Figure 2). The secondary endpoint MACCE occurred in 116 (7.1%) clopidogrel-treated patients and 68 (7.0%) ticagrelor-treated patients. There were no significant differences in MACCE (unadjusted HR, 1.02, and 95% CI, 0.75–1.37; IPTW-adjusted HR, 1.13, and 95% CI 0.83–1.55; Table 2; Figure 3), even in terms of the individual MACCE components (Table 2). The other secondary endpoint clinically significant bleeding (BARC ≥2) within 12 months occurred significantly less often in clopidogrel-treated patients than in ticagrelor-treated patients (27, 1.7%, vs. 31, 3.2%, respectively; unadjusted HR, 0.51, and 95% CI, 0.31–0.86; IPTW-adjusted HR, 0.42, and 95% CI, 0.25–0.69; Table 2; Figure 4).

**Table 2:** Risk for primary and secondary endpoints at 12 months after PCI.

**Events**	**Clopidogrel** **(*N =* 1,636)**	**Ticagrelor** **(*N =* 975)**	**Crude model**	**IPTW-adjusted cox regression model**	**Multivariate cox regression model**
NACCE: all-cause death, MI, stroke, stent thrombosis, urgent coronary revascularization, clinically significant bleeding (BARC ≥2)	138 (8.4%)	95 (9.7%)	0.86 (0.66 to 1.11)	0.86 (0.66 to 1.12)	0.85 (0.64 to 1.11)
MACCE: all-cause death, MI, stroke, stent thrombosis, urgent coronary revascularization	116 (7.1%)	68 (7.0%)	1.02 (0.75 to 1.37)	1.13 (0.83 to 1.55)	1.05 (0.77 to 1.44)
All-cause death	22 (1.3%)	10 (1.0%)	1.31 (0.62 to 2.78)	1.52 (0.68 to 3.42)	1.25 (0.57 to 2.72)
MI	11 (0.7%)	4 (0.4%)	1.64 (0.52 to 5.16)	1.69 (0.52 to 5.52)	1.65 (0.50 to 5.45)
Stroke	26 (1.6%)	9 (0.9%)	1.73 (0.81 to 3.70)	1.57 (0.75 to 3.32)	1.67 (0.75 to 3.69)
Stent thrombosis	20 (1.2%)	9 (0.9%)	1.33 (0.61 to 2.92)	1.42 (0.62 to 3.22)	1.30 (0.58 to 2.92)
Urgent coronary revascularization	63 (3.9%)	45 (4.6%)	0.83 (0.57 to 1.22)	0.95 (0.64 to 1.42)	0.94 (0.63 to 1.40)
Clinically significant bleeding (BARC≥2)	27 (1.7%)	31 (3.2%)	0.51 (0.31 to 0.86)	0.42 (0.25 to 0.69)	0.45 (0.26 to 0.80)

*BARC, Bleeding Academic Research Consortium; MACCE, adverse clinical and cerebral events; MI, myocardial infarction; NACCEs, net adverse clinical and cerebral events*.

Table 3 shows the results of stratified analyses. No significant interaction association was detected between the treatment condition and age (≥75 years and <75 years), sex, BMI (>30 and ≤ 30 kg/m^2^), or diabetes mellitus in terms of the primary or secondary endpoints.

**Table 3:** Risk for primary and secondary endpoints according to selected subgroups of study patients.

	**NACCE**		**MACCE**		**Clinically significant bleeding**	
**Variables**	**Adjusted HR (95% CI)**	***P* for interaction**	**Adjusted HR (95% CI)**	***P* for interaction**	**Adjusted HR (95% CI)**	***P* for interaction**
Age, years		0.807		0.321		0.451
≥75	0.77 (0.35 to 1.69)		1.93 (0.63 to 5.95)		0.22 (0.06 to 0.88)	
<75	0.88 (0.66 to 1.19)		1.02 (0.73 to 1.41)		0.53 (0.29 to 0.98)	
Gender		0.146		0.205		0.224
Male	0.72 (0.52 to 1.01)		0.91 (0.62 to 1.33)		0.40 (0.19 to 0.83)	
Female	1.28 (0.77 to 2.12)		1.76 (0.97 to 3.19)		0.55 (0.21 to 1.39)	
BMI, kg/m^2^		0.631		0.375		0.363
>30	0.58 (0.19 to 1.82)		1.71 (0.39 to 7.48)		0.33 (0.05 to 2.12)	
≤ 30	0.88 (0.66 to 1.17)		1.03 (0.74 to 1.42)		0.49 (0.27 to 0.88)	
Diabetes mellitus		0.648		0.287		0.558
Yes	0.76 (0.48 to 1.18)		0.82 (0.50 to 1.35)		0.54 (0.21 to 1.37)	
No	0.89 (0.63 to 1.27)		1.21 (0.80 to 1.83)		0.40 (0.20 to 0.82)	

*BMI, body mass index; MACCE, adverse clinical and cerebral events; NACCEs, net adverse clinical and cerebral events*.

## Discussion

Increases in the extension of average life expectancy have led to a gradual increase in the proportion of older individuals worldwide. The population of those aged ≥60 years is expected to reach two billion by 2050 (19). Studies have shown that morbidity and mortality due to ACS in older patients are higher than those in younger patients (20), posing a challenge and burden to medical and healthcare systems and highlighting the need for an optimal DAPT strategy to improve prognosis in older patients, a high-risk group. There are no explicit criteria to define older individuals, perhaps because aging is a continuous process and older patients only represent a minority in randomized clinical trials; we therefore applied a cutoff of 65 years, the lower age limit used for risk stratification (5, 6, 10, 11).

Based on the abovementioned considerations and rationale, we conducted the present real-world study to compare the clinical outcomes of clopidogrel and ticagrelor—a more potent P2Y12 inhibitor—uses in older patients with ACS aged ≥65 years who underwent PCI. Our main findings suggested that clopidogrel and ticagrelor had comparable net clinical benefits in these patients. Furthermore, clopidogrel was associated with a lower risk of clinically significant bleeding events than ticagrelor, without an increase in the risk of ischemic events. Our study thus provides evidence for clopidogrel as an effective alternative to ticagrelor in older patients.

Our main finding, the comparable effectiveness of ticagrelor and clopidogrel, is similar to that of a subgroup analysis in the TRITON-TIMI 38 trial (1), which showed that the more potent P2Y12 inhibitor prasugrel had no net clinical benefit in patients aged ≥75 years compared with clopidogrel (HR, 0.99; 95% CI, 0.81–1.21). Although prasugrel reduced ischemic events, concomitant high rates of major bleeding counterbalanced its net clinical benefit. Moreover, the POPular AGE trial reported similar results. POPular AGE is an open-label, randomized control trial that was conducted in 12 sites to compare the safety and efficacy of clopidogrel and ticagrelor or prasugrel in patients aged ≥70 years; the trial findings revealed that clopidogrel had a non-inferior net clinical benefit compared with ticagrelor (HR, 0.82; 95% CI, 0.66–1.03) (8). Although previous RCTs have supported clopidogrel use as an alternative to more potent P2Y12 inhibitors in older patients, this study, based on real-world data, reports evidence from an East Asian population.

An optimal DAPT strategy should balance the risk of ischemic and bleeding events. Aging is a common predictor for both risks, likely because of the fact that vascular aging is related to atherosclerosis (21), which increases levels of fibrinogen (22), alterations in liver enzymes and pharmacokinetics (23), and greater comorbidities. Owing to a larger reduction in ischemic events, guidelines recommend the more potent P2Y12 inhibitors ticagrelor and prasugrel over clopidogrel in patients with ACS (4). However, several previous studies have reported that older patients receiving more potent P2Y12 inhibitors have a higher risk of bleeding (24) and even life-threatening bleeding (e.g., fatal intracranial bleeding) than younger patients, which leads to earlier mortality and reduced net clinical benefit. Additionally, premature discontinuation due to minor bleeding also places patients at a high risk for ischemic events (25).

Reducing the bleeding risk associated with potent P2Y12 inhibitors without increasing the ischemic risk is, therefore, an essential consideration for the selection of DAPT in older patients. Our results showed that clopidogrel had a significantly lower incidence of clinically significant bleeding events (BARC ≥2) than ticagrelor (IPTW-adjusted HR, 0.42; 95% CI 0.25–0.69) in older patients. Although confounded by ambiguous definitions of older individuals and major bleeding events, a meta-analysis by Tomohiro et al. (26) that included nine RCT studies revealed that the potent P2Y12 inhibitors increased the risk of major bleeding events compared with clopidogrel in older patients (HR, 1.27; 95% CI 1.04–1.56). In addition, the POPular AGE trial not only reported the HR of the primary bleeding endpoint defined as PLATO major and minor bleeding (HR, 0.71; 95% CI, 0.54–0.94) while comparing clopidogrel with ticagrelor but also the HR of bleeding outcomes defined based on the other criteria, which includes BARC class 2 (HR, 0.65; 95% CI, 0.48–0.89), and classes 3 and 5 (HR, 0.61; 95% CI, 0.38–0.98), which are consistent with the finding of this study. In addition, a registry study that includes patients aged ≥80 years also reported similar findings—specifically, that ticagrelor was associated with a 48% higher risk of bleeding after MI than clopidogrel (27).

This study showed that clopidogrel and ticagrelor had a comparable risk of ischemic events in older patients in terms of the composite MACCE endpoint and also its individual components (all-cause death, MI, stroke, stent thrombosis, and urgent coronary revascularization). However, there is still considerable lack of clarity in terms of the risk of ischemic events due to potent P2Y12 inhibitors vs. clopidogrel among older patients. In 2016, Wang et al. (28) published a randomized controlled trial in the Chinese population, which demonstrated that compared with clopidogrel, ticagrelor reduced the risk of composite ischemic events (including cardiovascular death, MI, and stroke; HR, 0.473; 95% CI, 0.230–0.976) without increasing the risk of bleeding events (HR, 1.410; 95% CI, 0.717–2.774) among patients aged ≥65 years. However, their findings might be biased and not strongly representativeness, because it was a single-center study that involves only 200 patients. The PLATO trial suggested that ticagrelor reduced ischemic events compared with clopidogrel regardless of age (≥75 years vs. ≤ 75 years; *p* for association = 0.56), although no significant difference was reported in the ischemic events between the two groups in patients aged ≥75 years (7). Moreover, a registry study includes about 3,500 patients with ACS aged ≥75 years similarly reported that ticagrelor did not benefit all patients and that it failed to further reduce the incidence of MI in older patients (HR, 0.25; 95% CI, 0.1–1.1; *p* = 0.072) (29).

Although guidelines recommend that potent P2Y12 inhibitors are superior to clopidogrel in patients with ACS who underwent PCI in terms of further reduction in ischemic events, the use of ticagrelor in older Chinese patients has been low. Only 37.3% patients received ticagrelor in this study, which was consistent with the previous studies conducted in Chinese populations (30). There are several possible reasons for this low utilization of ticagrelor. First, the “East Asian Paradox” study published in 2014 (31) suggested that East Asian patients have a significantly higher risk of bleeding with a similar or lower risk of ischemia compared with their Caucasian counterparts. Hence, cardiologists prefer clopidogrel, particularly for older patients, because of the lower risk of major bleeding than that noted in the case of ticagrelor. Second, clopidogrel has fewer other side effects (e.g., dyspnea) than ticagrelor (32). Third, clopidogrel has been marketed earlier in China with better accessibility and affordability than ticagrelor.

The high prevalence of unstable angina in this study (approximately 82.4%) was inconsistent with that noted in studies conducted in other countries or clinical centers (nearly 7.5–30.7%) (32–34). A likely reason for such high unstable angina prevalence in this study is also one of its limitations; this was a single-center study and, therefore, might not have been a broad representative of the patient population, and the findings were probably influenced by the expertise of the clinical center. Natural diversity among populations and disease conditions also might have led to differences in the proportion of unstable angina. However, the characteristics and prevalence of risk factors in our Asian cohort, such as smoking status and medical history, were similar to that of other studies, which includes those involving other ethnicities; this supports the generalizability of our results (35, 36).

The other important limitations of our study are mentioned below. (1) The study design was observational and retrospective. The selection of P2Y12 inhibitors was at the discretion of cardiologists, which might have led to selection bias and significant differences in covariates between the two groups. Although IPTW was used to minimize baseline differences, not all covariates were considered; furthermore, the study's retrospective design might have led to potential bias. (2) Medication compliance was not evaluated, and this might have influenced the results because such compliance might be lower in older patients than in younger patients. (3) Because prasugrel is not yet licensed in China, ticagrelor was the only potent P2Y12 inhibitor used in this study. However, a previous meta-analysis showed that ticagrelor use has ischemic and bleeding risks similar to those associated with prasugrel use in patients with ACS (37). (4) Individual differences in the metabolism of clopidogrel might have affected the clinical outcomes of patients receiving clopidogrel. Further subgroup analyses based on CYP2C19 genotypes or platelet function are warranted to validate our findings. (5) Finally, as mentioned previously, this study enrolled patients from a single center and thus might not be broadly representative of all patients; this justifies the need to conduct a multicenter trial to further validate our findings.

## Conclusions

The present observational study showed that in patients with ACS aged ≥65 years, clopidogrel and ticagrelor had comparable net clinical benefits. Additionally, clopidogrel was associated with a significantly lower risk of major bleeding than ticagrelor without an increase in ischemia risk. Our findings suggest that clopidogrel is a useful alternative to ticagrelor in older patients.

## Data Availability Statement

The raw data supporting the conclusions of this article will be made available by the authors, without undue reservation.

## Ethics Statement

The studies involving human participants were reviewed and approved by Ethics Committee of Anzhen Hospital. The patients/participants provided their written informed consent to participate in this study.

## Author Contributions

YL and XS: conception and design. YL: administrative support. YZ, JH, and YW: collection and upload of data. YZ, JH, WP, and ZF: data analysis and interpretation. YZ and WP: manuscript writing. All authors contributed to the article and approved the submitted version.

## Conflict of Interest

The authors declare that the research was conducted in the absence of any commercial or financial relationships that could be construed as a potential conflict of interest.

## Publisher's Note

All claims expressed in this article are solely those of the authors and do not necessarily represent those of their affiliated organizations, or those of the publisher, the editors and the reviewers. Any product that may be evaluated in this article, or claim that may be made by its manufacturer, is not guaranteed or endorsed by the publisher.
